# Etiologies and clinical characteristics of macular hole: An 8-year, single-center, retrospective study

**DOI:** 10.1097/MD.0000000000037878

**Published:** 2024-08-09

**Authors:** Huifang Yue, Chenyu Liu, Yunda Zhang, Lijuan Zhang, Zhao Gao, Tao Ma, Ximei Zhang

**Affiliations:** a Shanxi Eye Hospital, Taiyuan, China; b Shanxi Medical University, Taiyuan, China.

**Keywords:** idiopathic macular hole, myopic macular hole, traumatic macular hole, clinical characteristics, baseline visual acuity

## Abstract

To investigate the etiologies and clinical characteristics of full-thickness macular hole (FTMH) cases at Shanxi Eye Hospital in North China. Patients diagnosed with FTMH who underwent surgery from 2012 to 2020 were included, and the etiologies and clinical features of MH types were analyzed in an 8-year, cross-sectional, retrospective study. A total of 752 cases (776 eyes) were analyzed. The top 3 subtypes of MH were idiopathic (IMH, 64.4%), myopic (MMH, 21.1%) and traumatic (TMH, 3.7%) MH. Among these, there were significant differences in sex, age, and baseline best-corrected visual acuity (BCVA) distributions. Females predominated in the IMH and MMH groups, while males predominated in the TMH group. The IMH onset age was older than the MMH and TMH onset ages. Baseline BCVA in the IMH (*Z* = 8.9, *P* < .001) and the other group (*Z* = 4.0, *P* < .001) was significantly better than that in the MMH group. In the IMH group, females were younger, had a shorter axial length (AL), and had a worse baseline BCVA than males, while in the MMH group, there were no significant sex differences. Multivariate correlation analysis showed that a smaller hole diameter in IMH, no retinal detachment in MMH, and a younger age in TMH may result in better baseline BCVA. The most common MH etiologies were IMH, MMH and TMH, which contributed to differences in clinical features. Females predominated in the IMH and MMH groups, and the onset of MMH occurred 6.5 years earlier than the onset of IMH. Therefore, early fundus monitoring in females and high myopia patients is helpful for the early detection and treatment of MH.

## 1. Introduction

Full-thickness macular hole (FTMH) is one of the main causes of central visual impairment,^[1]^ and the most common etiology is idiopathic macular hole (IMH) associated with vitreous macular traction syndrome. Other causes include high myopia, trauma, diabetic retinopathy (DR), a history of vitrectomy, etc.^[2–4]^ Previous studies have shown that the prevalence of MH is 0.17% and gradually increases with age; the prevalence of bilateral MH is 0.026%.^[5]^ In a retrospective study in Tasmania, Australia, the incidence of MH was 4.05 per 100,000 persons/yr, and the highest prevalence was in the 70 to 79 years age group. IMH, traumatic MH (TMH), and myopic MH (MMH) accounted for 87.1%, 5.4%, and 2.0% of the total cases of MH.^[6]^ Previous studies^[5,7,8]^ on the clinical features of MMH and TMH have been relatively rare compared to studies on IMH, and the results have shown that the incidence rates of IMH and MMH are higher in females, while the incidence of TMH is higher in young males. Studies^[9–11]^ have found that the factors affecting baseline vision include hole size in IMH and MMH with retinal detachment (RD); no results from TMH-related studies have been reported.

It is well known that East Asia has the largest number of people with myopia worldwide, and the prevalence of high myopia ranges from 6.8% to 21.6%, while it ranges from 1% to 4% elsewhere.^[12]^ This difference may result in different composition ratios of diverse MH types in published articles, and literature comparing the clinical features of all 3 types is scarce.^[4]^ To date, we have not found any relevant studies from China. Therefore, the aim of this study was to explore the etiologies and clinical characteristics of MH and to analyze related factors affecting baseline vision at Shanxi Eye Hospital in North China.

## 2. Methods

### 2.1. Inclusion and exclusion criteria for MH

This study was conducted at Shanxi Eye Hospital, which is the only tertiary eye hospital in Shanxi Province, North China, and was approved by the ethnic committee of Shanxi Eye Hospital. The study protocol adhered to the tenets of the Declaration of Helsinki. FTMH patients who were admitted to the hospital and underwent surgery in our hospital from October 2012 to October 2020 were included. According to etiology, they were classified into 4 groups: IMH, MMH, TMH and other. The inclusion criteria for MMH were refraction > 6.00 DS or axial length (AL) ≥ 26.0 mm, with or without RD. For those with RD, there was only one MH, and the extent did not exceed the retinal vascular arch. Patients with a history of ocular trauma were included in the TMH group regardless of immediate or gradual visual loss. The other group included those with MH due to all other recorded causes, such as vitrectomy, history of DR, retinal vein occlusion, glaucoma, laser photocoagulation or intraocular injection. Patients were excluded when they had peripheral RD caused by peripheral retinal degeneration or refractive media opacity. The priority order of enrollment was Other > TMH > MMH > IMH.

### 2.2. Preoperative parameters and examinations

The collected data included age at onset; sex; affected eye; duration of symptoms; preoperative intraocular pressure; AL; preoperative best-corrected visual acuity (BCVA); ocular surgery history; general state; and diameter of the hole, defined as the minimum diameter of the aperture (using Spectralis OCT, Heidelberg, Germany). Visual acuity was expressed using the logarithm of the minimum angle of resolution (logMAR), as referenced in previous studies,^[13]^ and counting fingers and hand movement were defined as 2.0 and 3.0 DS, respectively.

### 2.3. Statistical analysis

All data were statistically analyzed using SPSS 21.0 (Armonk, NY). In the descriptive analysis, continuous variables are expressed as means ± standard deviations (SDs) or medians and quartiles, and categorical data are expressed as numbers and proportions. The chi-square test was used to analyze differences among categorical data. For continuous variables, if the variables conformed to a normal distribution, we used a t test or ANOVA; otherwise, we used the Mann–Whitney *U* test or Kruskal–Wallis test to compare differences. Spearman correlation analysis was used to evaluate relationships among factors. Multivariate linear regression analysis assessed factors affecting baseline vision. A *P* value <.05 was considered significant.

## 3. Results

### 3.1. The classification of MHs etiology

**Figure 1.**
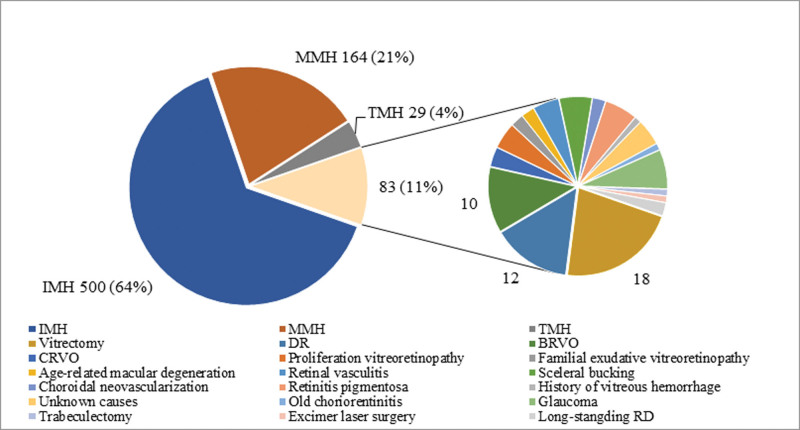
Proportions of eyes and potential causes by type of MHs.

In total 776 eyes (752 patients) were enrolled. The eyes were classified into 4 groups, as shown in Figure 1. IMH, MMH, and TMH were the top 3 subtypes. All TMHs except 3 laser injury cases were caused by closed-globe trauma. In the other group, possible causes included vitrectomy, scleral buckling, trabeculectomy, branch/central retinal vein occlusion (BRVO/CRVO), proliferative vitreoretinopathy, DR, retinal vasculitis, retinitis pigmentosa, familial exudative vitreoretinopathy, history of vitreous hemorrhage, glaucoma, and unknown causes. Among these patients, 21.7% had a history of vitrectomy, and 14.5% had Dr IMH and MMH occurred in 51 cases (10.6%) and 16 cases (10.1%) during the observation period, respectively.

### 3.2. Clinical characteristics of MHs

**Figure 2.**
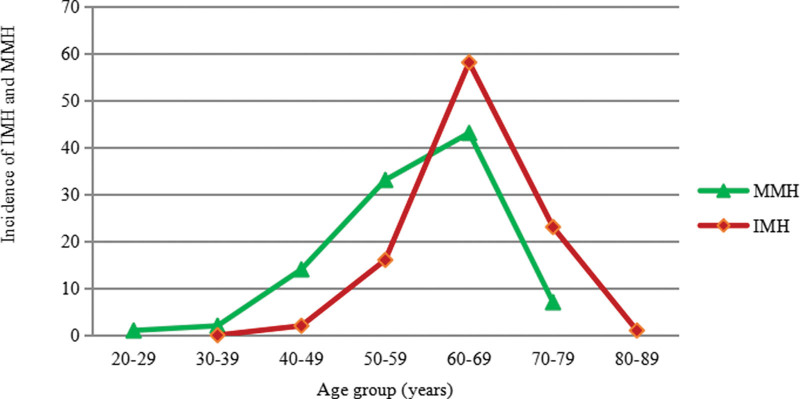
The incidence of IMH and MMH in different age groups. IMH = idiopathic macular hole, MMH = myopic macular hole.

The clinical features in the IMH, MMH, TMH and other subtypes are shown in Table 1. In terms of the male-to-female ratio of affected eyes, the proportion of males was higher in the TMH group, while the proportion of females was higher in the other groups. The IMH group and other group had better BCVA than the MMH group. Increasing age at onset was as follows: IMH > MMH > TMH. Age at onset in the MMH group was 6.5 years earlier than that in the IMH group, and the age at onset in the other group was comparable to that in the MMH group. The incidence rates of IMH and MMH were different among the different age ranges, as shown in Figure 2. Before the sixth decade, the incidence of MMH was higher than that of IMH, but after the sixth decade, MMH had a lower incidence.

**Table 1:** Comparisons of clinical characteristics among MH cases.

	IMH	MMH	TMH	Others	*P*	χ^2^
Patients/Eyes (n)	482/500	158/164	29/29	83/83		
Males/Females (n)	98/384	33/125	25/4^a^^,^^b^	22/61^c^	<.001*	66.8^d^
OS/OD (n)	242/258	76/88	20/9	46/37	.091	6.4^d^
Age (yr)	64.9 ± 6.8	58.5 ± 9.0^a^	34.3 ± 13.8^a^^,^^b^	57.4 ± 14.3^a^^,^^c^	<.001*	143.6^e^
Duration (mo)	3.0 (2.0, 6.0)	3.0 (1.0, 12.0)	3.0 (1.4, 10.3)		.670	0.8^e^
Diameter of hole (μm)	518.5 ± 197.4	572.4.0 ± 261.1	609.8 ± 298.4		.053	5.9^e^
Baseline BCVA logMAR	1.09 ± 0.47	1.66 ± 0.76^a^	1.46 ± 0.88	1.31 ± 0.36^b^	<.001*	80.8^e^

BCVA = best-corrected visual acuity, IMH = idiopathic macular hole, MMH = myopic macular hole, TMH = traumatic macular hole.

* *P* < .05.

a *P* < .05 compared to IMH.

b *P* < .05 compared to MMH.

c *P* < .05 compared to TMH.

d Kruskal–Wallis.

e Pearson’s chi-square test.

### 3.3. Statistics analysis of clinical characteristics in different MHs

**Figure 3.**
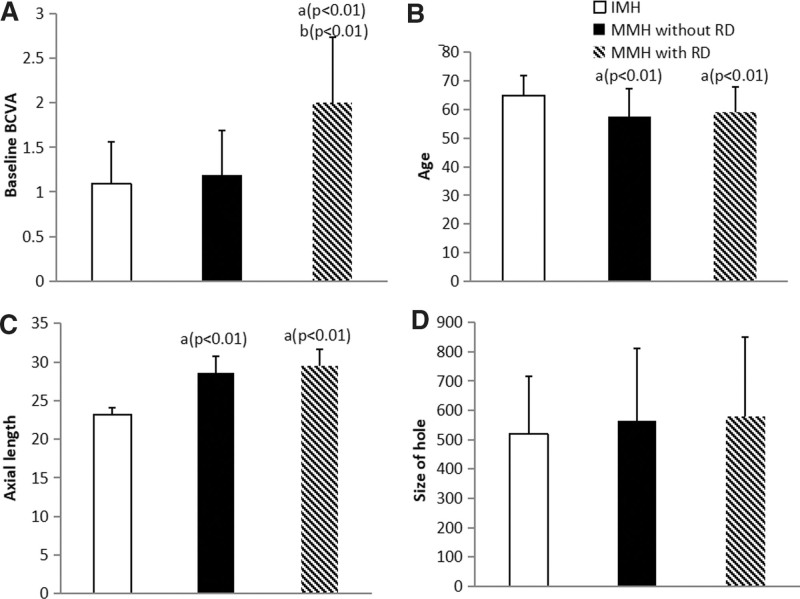
Comparisons of the clinical features between IMH and MMH patients with and without RD. IMH = idiopathic macular hole, MMH = myopic macular hole, RD = retinal detachment.

Regarding the differences in epidemiological characteristics between the sexes (Table 2), we found that females were significantly younger (*Z* = 3.5, *P* < .001), had a worse baseline BCVA (*Z* = 2.6, *P* = .010) and had a shorter AL (*Z* = 7.0, *P* < .001) than males, while there were no differences in hole diameter or duration of IMH. There were no differences in the proportions of MMH and TMH between the sexes. In the MMH group, patients with RD had a longer AL (*Z* = −2.3, *P* = .021), shorter duration of symptoms (*Z* = 3.2, *P* = .01) and worse BCVA (*Z* = −6.9, *P* < .001) than those without RD. After comparing the data of patients with IMH and MMH with and without RD (Fig. 3), no difference was found in the baseline BCVA between IMH and MMH patients without RD.

**Table 2:** Comparisons of clinical features between sexes in MH patients.

	IMH			MMH			TMH		
	Males	Females	*P*	Males	Females	*P*	Males	Females	*P*
Eyes (n)	101	399		35	129		25	4	
Age (yr)	67.2 ± 6.5	64.5 ± 6.5	<.001_a_*	59.9 ± 11.2	58.1 ± 8.4	.074_a_	31.8 ± 15.4	41.5 ± 14.5	.252_b_
∫Duration (mo)	3.0 (2.0, 6.0)	3.0 (2.0, 6.0)	.602_a_	2.0 (0.6, 6.0)	3.0 (1.0, 12.0)	.311_a_	3.0 (1.4, 3.0)	7.0 (0.68, 453.0)	.647_a_
AL (mm)									
mean ± SD	23.77 ± 0.90	23.09 ± 0.96	<.001_a_*	28.99 ± 2.17	29.14 ± 2.19	.745_a_	24.14 ± 1.51	26.78 ± 2.45	.027 _a_*
Not recorded (n)	7	15		3	9		3	1	
Baseline BCVA	0.98 ± 0.41	1.12 ± 0.48	.010*_a_	1.67 ± 0.90	1.66 ± 0.72	.692_a_	1.40 ± 0.81	1.83 ± 1.41	.482_a_
Size of hole (μm)	488.6 ± 188.2	526.1 ± 200.0	.088_b_	568.4 ± 307.0	573.5 ± 248.5	.884_a_	642.0 ± 298.1	417.0 ± 140.7	.095_a_
With/Without RD (n)				20/15	75/54	.916_c_			

BCVA = best-corrected visual acuity, IMH = idiopathic macular hole, MMH = myopic macular hole, RD = retinal detachment, TMH = traumatic macular hole.

* *P*<.05, ∫median (quartiles).

a Mann–Whitney *U* test.

b independent-samples *t* test.

c Pearson’s chi-square test.

### 3.4. The factors affecting baseline best-corrected visual acuity

The factors related to preoperative BCVA in MH patients are shown in Table 3. The factors affecting BCVA in the IMH group were sex, hole size and symptom duration, but the BCVA differed between only the ≤1 month and 3 to 6-month duration groups. After adjusting for possible confounding factors, only hole size was significantly associated with BCVA (*R* = 0.386, *P* < .001, 95% CI 0.313–0.463). Moreover, there was a significant correlation between symptom duration and hole size in the IMH group (*R* = 0.303, *P* < .001, 95% CI 0.223–0.387). Spearman correlation analysis showed that both AL and RD were correlated with BCVA in the MMH group, while after controlling for possible confounding factors, RD was the only factor correlated with a worse BCVA (*R* = −0.491, *P* < .001, 95% CI −0.590 to −0.374). Multivariate correlation analyses of AL, hole size, age and BCVA in the TMH group showed that there was a significant correlation between only age and BCVA (*R* = 0.446, *P* = .025, 95% CI 0.051–0.714).

**Table 3:** Factors affecting preoperative BCVA in the IMH and MMH groups.

	IMH	*P*	Statistics	MMH	*P*	Statistics
Males/females (eye, n)	101/399	.010*	*U* = 23459.0 *Z* = 2.6_a_	35/129	.692	*U* = 2354.4 *Z* = 0.4_a_
Age (yr)	64.9 ± 6.8			58.5 ± 9.0		
<49	10 (2.0%)	.145	χ^2^ = 6.8_b_	27 (16.5%)	.410	χ^2^ = 2.9_b_
50–59	77 (15.4%)			56 (34.1%)		
60–69	290 (58.0%)			68 (41.5%)		
79–79	116 (23.2%)			13 (7.9%)		
≥80	7 (1.4%)					
Duration (mo)						
≤1	111 (22.2%)	∫.004*	χ^2^ = 17.1_b_	54 (32.9%)	.531	χ^2^ = 4.1_b_
1–3	154 (30.8%)			37 (22.6%)		
3–6	132 (26.4%)			25 (15.2%)		
6–12	70 (14.0%)			28 (17.1%)		
12–36	27 (5.4%)			12 (7.3%)		
>36	6 (1.2%)			8 (4.9%)		
Size of hole (μm)	518.5 ± 197.4	<.001*	*R* = 0.419_c_	572.4.0 ± 261.1	.047*	*R* = 0.155_c_
With/Without RD (n)				95/69	<.001*	*U* = 1246.5 *Z* = 6.9_a_

BCVA = best-corrected visual acuity, IMH = idiopathic macular hole, MMH = myopic macular hole, RD = retinal detachment, TMH = traumatic macular hole.

* *P* < .05, ∫except for the difference between the ≤1 and 3 to 6-month groups (*P* = .028), no differences were observed between the other groups.

a Mann–Whitney *U* test

b Kruskal–Wallis test.

c Spearman’s rank correlation analysis.

By multivariate linear regression analysis, baseline vision was associated with hole size (*P* < .001, 95% CI 0.001–0.001) and gender (*P* = .011, 95% CI 0.030–0.222), hole size increased by 100um, and baseline visual acuity was increased by 0.1, and baseline visual acuity was worse in women than in men. While analysis showed that worse MMH baseline visual acuity was associated with RD (B = 0.833, *P* < .001, 95% CI 0.624–1.042).

## 4. Discussion

The present 8-year study analyzed the etiologies and epidemiological characteristics of MH cases and compared the discrepancies in age and sex proportions in the IMH, MMH and TMH groups, as well as the different factors affecting baseline BCVA among these groups.

In previous epidemiological investigations in Norway and Australia^[6,9]^ the proportions of IMH were larger than that in this study, accounting for 85.9% and 87.1%, and the male-to-female ratios were 1:2.2 and 1:2, respectively, but in other retrospective clinical reports including surgical patients, the sex ratio was comparable to ours, at nearly 1:4.^[14,15]^ Additionally, in the Norway and Australian studies,^[6,9]^ MMH accounted for only 1% to 2%, while MMH accounted for 21.1% in our study. The reason for this difference is probably that we included MMH with RD (MHRD), which occurs in approximately 57.9% of all MMH cases. However, when MHRD cases were excluded, the proportion of MMH was still nearly 10% higher than that in previous reports. The higher prevalence of myopia in East Asia, including China, is attributed to different MH proportions.^[12,16]^ In a previous study^[9]^ in surgical patients, TMH accounted for 3% of all MH cases, which is consistent with that in our study, but in another study^[17]^ in MH patients, TMH accounted for 5% to 8.2%; moreover, those who were <24 years old and had a hole diameter of <0.2 DD had a greater chance of spontaneous closure.^[17]^ Therefore, we included only surgery patients with a significant decrease in BCVA or a trend of gradual enlargement of the hole during follow-up. TMH accounted for the second largest MHs^[7]^; however, in consideration of the MH proportions in our study and spontaneous closure in TMH, the accuracy of such results needs to be further verified.

The IMH group was older than the MMH and TMH groups, and the ages at onset of the 3 types of MHs may be related to their underlying pathogeneses. Both IMH and MMH are complications of posterior vitreous detachment (PVD), which is the consequence of the interaction between vitreous liquefaction and progressive weakening of vitreoretinal adhesion.^[18,19]^ In general, the posterior vitreous cortex initially detaches at the paramacular area and extends to the perifoveal area and then to the optical disc. Finally, complete PVD develops, and this inevitable process changes with age.^[18–21]^ IMH is caused by vitreomacular traction, which is characterized by aberrant PVD and accompanied by anatomic distortion of the fovea, whereas secondary MH is caused by pathologies other than vitreomacular traction.^[1]^ Axial elongation and the formation of posterior scleral staphyloma in high myopia accelerate vitreous liquefaction and increase its instability, resulting in abnormal PVD that is more likely to progress to MH; the greater the degree of refraction and the longer the AL, the earlier the PVD occurs.^[22,23]^ Although axial elongation contributed to the earlier occurrence of MMH, there was no correlation between age and AL in our study. Furthermore, in addition to the effect of PVD on the development of FTMH, lower concentrations of collagen, protein and hyaluronic acid can induce MH development.^[24]^

The exact mechanism of TMH following blunt trauma is still controversial, and it is generally believed that blunt trauma leads to foveal tissue loss caused by anteroposterior vitreous traction in the fovea. A sudden decrease in the globe’s anterior–posterior diameter causes equatorial expansion of the globe, resulting in horizontal and tangential forces and splitting of the retinal layers at the fovea.^[7,25,26]^ While Rossi et al^[26]^ found that TMH also occurred in nonvitreous eyes, their study revealed that damp shockwaves were also responsible for trauma-related retinal lesions. Accidental high-power laser-induced MH is caused mainly by rapid photothermal damage or photodisruptive mechanisms.^[27]^

With respect to age in IMH patients, the results of various epidemiological investigations are inconsistent, and average ages range from 56.2 to 70.2 years.^[6,9,28]^ A respective study^[4]^ of different types of MHs found that the mean age of MMH patients was 42 years, which was younger than that in our study. The rate of onset of both IMH and MMH increased with age, which is consistent with the change in PVD. The area of vitreous macular adhesion gradually decreases after 30 years, the stress on the fovea may increase with decreasing adhesion area, and the incidence of partial PVD with sustained PVD peaks in the sixth decade.^[29]^ Therefore, the age at onset of IMH is approximately 60 years old. PVD studies on MMH have shown that it generally occurs earlier that IMH,^[20,23]^ but the exact time is not yet known. In our study, the age at onset of MMH was 6.5 years younger than that of IMH, which may indicate that PVD in those with high myopia occurred almost 6.5 years earlier than PVD in those without myopia. Ali et al^[28]^ revealed that age was an independent risk factor for IMH, yet the proportions of both MMH and IMH showed gradual increases with age in our study, so it may also be an important risk factor for the occurrence of MMH. TMH is more common in young males, since ocular trauma mostly occurs while playing sports or during work-related accidents.^[7,25]^

Regarding onset in different sexes, females had a higher incidence and younger age at onset than males in the IMH and MMH groups, although there was no statistically significant difference in age at onset of MMH. In females, decreased estrogen affects connective tissues and causes the acceleration of vitreous liquefaction, accelerating the development of PVD and the decline in the vitreomacular adhesion area, ultimately leading to a higher incidence and earlier onset in females than in males.^[30–32]^ Previous studies^[9,15]^ on IMH found that males had longer AL than females, and there was no difference in baseline BCVA between sexes. The reason for the worse baseline BCVA in females in our study could be that the average hole diameter was larger than that in males, although this difference was nonsignificant. Similarly, Steel et al^[33]^ noted that females tended to have larger hole sizes than males. In contrast with IMH, MMH was not associated with differences in any of the preoperative parameters between the sexes. AL was different between the sexes in the TMH group because 2 of 3 females with retained axial data had high myopia.

Ghoraba et al^[4]^ observed BCVA in different MH-type groups and found no difference in baseline BCVA, which is inconsistent with the results of our study. This might be due to the exclusion of MHRD cases in their study. After removing the MHRD cases in our study, the BCVA difference between the MMH and IMH group was nonexistent, in accordance with the aforementioned studies. Our study found that RD was an important factor affecting BCVA in the MMH group. In the TMH group, due to the different causes and pathogeneses of ocular injury, lesions such as commotio retinae, choroidal rupture, or vitreous hemorrhage might have contributed to the BCVA.^[25,34]^ Ultimately, all lesions lead to uncertain visual function.

According to the multivariate analysis, in the IMH group, the smaller the hole was, the better the maintenance of baseline BCVA and the shorter the duration of symptoms was; however, the duration of symptoms had no significant correlation with BCVA, in accordance with previous reports.^[9,10]^ A better baseline BCVA was more likely to be achieved in eyes without RD in MMH patients, and the longer the AL was, the greater the probability of RD. The results of previous studies have shown that the occurrence of RD is related to AL.^[12]^ It was difficult to determine the correlation between a shorter symptom duration and MHRD due to inaccurate complaint durations and the compensatory effect of vision in the contralateral eye. In the TMH group, since the severity of trauma and fundus damage other than MH were hard to evaluate, it was not possible to determine the correlation between age and BCVA.

This study analyzed the etiologies and epidemiological characteristics of MH in North China. To our knowledge, this is the first 8-year study focusing on comparisons of clinical characteristics and factors influencing baseline BCVA among IMH, MMH and TMH patients. Unlike previous studies, our study had a relatively large sample size and a long study period, and included MMH patients with MHRD, with detachment limited to within the vascular arch. The current study still had many limitations. This was a retrospective study conducted in one center, and some AL data were missing. Fortunately, the small number of missing cases did not affect the results of the study. Our study included only patients who underwent surgery and did not contain observational cases, so it might have deviation in the ranking of the main etiologies. The spontaneous closure rate in TMH might be higher than those in IMH and MMH, reaching as high as 50% in children, as Miller et al^[34]^ described. Despite this, the number of TMH cases was smaller than the numbers of MMH and IMH cases.

Our data demonstrated that the most common subtypes of MH were IMH, MMH and TMH, and MMH accounted for 21.1% of all MMH cases, which was higher than those in previous studies. Different pathogeneses of the 3 types of MH cause significant differences in age at onset, the sex distribution and BCVA. In addition to age, female sex was a risk factor for IMH and MMH. The age at onset in the MMH group was nearly 6.5 years younger than that in the IMH group. Therefore, early monitoring of the fundus condition in myopic eyes is necessary for the early detection and interventional treatment of lesions.

## Acknowledgments

The authors thank Dr Zhigang Yuan for his support in collecting data and Dr Xiaohong Gao and Ping Wang for their valuable comments.

## Author contributions

**Data curation:** Huifang Yue, Yuexin Shi, Chenyu Liu.

**Formal analysis:** Yunda Zhang, Zhao Gao, Lijuan Zhang.

**Funding acquisition:** Yunda Zhang, Ximei Zhang.

**Methodology:** Huifang Yue, Ximei Zhang.

**Resources:** Ximei Zhang.

**Software:** Chenyu Liu.

**Writing – original draft:** Huifang Yue, Yuexin Shi.

**Writing – review & editing:** Huifang Yue.
